# DNase II Can Efficiently Digest RNA and Needs to Be Redefined as a Nuclease

**DOI:** 10.3390/cells13181525

**Published:** 2024-09-11

**Authors:** Jingyun Zhuang, Xinmei Du, Kehan Liu, Jing Hao, Haoyu Wang, Ran An, Xingguo Liang

**Affiliations:** 1 State Key Laboratory of Marine Food Processing & Safety Control, College of Food Science and Engineering, Ocean University of China, Qingdao 266404, China; zhuangjingyun@stu.ouc.edu.cn (J.Z.); duxinmei@stu.ouc.edu.cn (X.D.); 11240711001@stu.ouc.edu.cn (K.L.); haojing2127@163.com (J.H.); why1614343632@163.com (H.W.); 2 Laboratory for Marine Drugs and Bioproducts, Qingdao Marine Science and Technology Center, Qingdao 266404, China

**Keywords:** DNase II, RNA digestion, hydrolysis, biological functions, sequence specificity

## Abstract

DNase II, identified in 1947 and named in 1953, is an acidic DNA endonuclease prevalent across organisms and crucial for normal growth. Despite its expression in nearly all human tissues, as well as its biological significance, DNase II’s detailed functions and corresponding mechanisms remain unclear. Although many groups are trying to figure this out, progress is very limited. It is very hard to connect its indispensability with its DNA cleavage activity. In this study, we find that DNase II secreted to saliva can digest RNA in mildly acidic conditions, prompting us to hypothesize that salivary DNase II might digest RNA in the stomach. This finding is consistent with the interesting discovery reported in 1964 that RNA could inhibit DNase II’s activity, which has been largely overlooked. This RNA digestion activity is further confirmed by using purified DNase II, showing activity to digest both DNA and RNA effectively. Here, we suggest redesignating DNase II as DNase II (RNase). The biological functions of DNase II are suggested to recycle intracellular RNA or digest external nucleic acids (both RNA and DNA) as nutrients. This discovery may untangle the mystery of DNase II and its significant biofunctions.

## 1. Introduction

DNase II was first identified in 1947 [1] in *Drosophila melanogaster*. Despite its definite role in degrading DNA within lysosomes or phagocytic cells, its detailed functions and mechanisms remain largely unknown. DNase II is an acidic endonuclease that produces cleavage products with 3′-PO_4_ and 5′-OH ends, which cannot be directly used by ligases [2,3]. It has been found to be widely distributed across various mammalian tissues [4,5,6], predominantly located in lysosomes [7,8]. Research by Krieser revealed the high expression of DNase II in salivary glands, and Toshihiro confirmed that there are significant amounts of DNase II in saliva [6,9]. Additionally, DNase II has been detected in the human gastric mucosa [10]. These findings suggest that DNase II, active under acidic conditions, might digest DNA in the stomach.

Unlike common nucleases like DNase I and RNase A, DNase II has activity optimally at a pH of 4.5–5.5 and does not require divalent or even monovalent cations for hydrolysis [11,12]. Single-stranded DNA (ssDNA) is more readily digested by DNase II compared to double-stranded DNA (dsDNA). The digestion of dsDNA typically begins by breaking one strand to form nicks so that dsDNA dissociates into single strands partly and then digests progressively [13,14,15]. Generally, DNase II has no significant sequence specificity [11], although certain species, including Caenorhabditis elegans, *Drosophila melanogaster*, and cattle, exhibit a preference for substrates containing the AGA↓GGA sequence (where the arrow indicates the phosphodiester bond cleavage site) [16]. The secondary structure of DNA, but not its sequence, influences DNase II’s cleavage efficiency more greatly [17].

In 1964, researchers discovered that RNA molecules such as t-RNA, r-RNA, polyU, and polyA/polyU complexes could reduce the degradation rate of DNA by DNase II, suggesting a nonspecific interaction between RNA and DNase II [18,19]. As is well known, RNA’s 2′-OH group typically facilitates the hydrolysis of its own phosphodiester bonds, making RNA more susceptible to degradation. For instance, RNase A causes RNA digestion to produce an unstable 2′,3′-cyclic phosphate intermediate, which is then hydrolyzed to 3′-phosphate nucleic acid [20,21]. Thus, evolving an enzyme that only digests ssDNA to produce 3′-phosphate products without degrading RNA is difficult and unreasonable. Accordingly, we hypothesize that RNA’s inhibitory effect on DNase II’s activity may stem from DNase II’s preferential degradation of RNA.

The widespread expression of DNase II suggests it has an important role, but there is rarely foreign DNA in mammalian cells. Scientists have been exploring its function for a long time [22]. For example, researches have shown that DNase II can efficiently digest DNA within cells [23,24], especially the DNA of apoptotic cells in mice [25]. DNase II knockout mouse embryos exhibit many nucleated red blood cells in their peripheral blood and cannot be born normally [24]. DNase II also plays roles in various developmental processes in mammals, including embryogenesis [26], final erythropoiesis [24], lens cell differentiation [27,28], and phagocytosis-mediated DNA degradation [24]. The absence of DNase II in phagocytes leads to the accumulation of discarded DNA [14,23,24,29,30,31,32], significantly impacting development and immunity [25,33]. However, the exact mechanism by which DNase II performs its biological functions remains unclear. Scientists remain puzzled about how cells and tissues utilize DNase II [11].

In this study, we discovered that DNase II can digest RNA very efficiently, supposing that DNase II can digest RNA under acidic conditions and probably use it as a nutrient. This was further confirmed by the fact that RNA degradation by DNase II was significantly inhibited by copper ions (while RNase A’s inhibitor did not inhibit it). The characteristics of DNase II’s RNA degradation and its substrate preferences were also investigated.

## 2. Materials and Methods

### 2.1. Materials

T4 DNA ligase (EL0014), DNA Ladder (SM1211), Fast AP (EF0654), dNTP (R0192), GMP, ATP, UTP, CTP, and GTP (R0481) were purchased from Thermo Scientific (Pittsburgh, PA, USA). T7 RNA polymerase (30223-1) was purchased from Biosearch Technologies, Inc (TW11 0LY, Middlesex, UK). DNase II (from pig spleen, S10074) was purchased from Shanghai Yuanye Bio-Technology Co., Ltd. (Shanghai, China) and was dissolved in pure water to a concentration of 200 U/µL (including 0.05 M DTT and 50% Glycerol). T4 DNA ligase and exonucleases of XRN-1 (M0338L) and EXO T (M0265L) were purchased from New England Biolabs Inc. (Ipswich, MA, USA). Ultra GelRed (GR501-01) was purchased from Vazyme (Nanjing, China). RNase inhibitor (RK21401), DNA Polymerase I, Large (Klenow) Fragment (RK20525), DNase I (RK20549), and T4 polynucleotide kinase (T4 PNK, RK20524) were purchased from ABclonal Biotech Co., Ltd. (Wuhan, Hubei, China). Salmon sperm DNA (10~1000 bp) was purchased from Tokyo Pharmaceutical Factory Limited (Tokyo, Japan) and was dissolved in pure water to a concentration of 16.0 g/L. Nucleic extraction (phenol/chloroform/isoamyl alcohol volume ratio of 25:24:1, pH ≥ 7.8 for DNA, EX0128; pH ≤ 5.0 for RNA, EX0105) was purchased from Jinclone (Beijing, China) Biotechnology Co., Ltd. (Beijing, China). Nucleic extraction (chloroform/isoamyl alcohol volume ratio of 24:1, P1014) and yeast RNA (<100 nt, G8670-5) were purchased from Beijing Solarbio Science & Technology Co., Ltd. (Beijing, China). Yeast RNA was dissolved in pure water to a concentration of 16.0 g/L. All DNAs used in this study (see Supplemental Table S1 for sequences) were purchased from Sangon Biotech (Shanghai) Co., Ltd. (Shanghai, China). Fluorescent modified RNA and other RNAs were purchased from Azenta, Inc. (Burlington, MA, USA). All other chemicals were from Sigma-Aldrich (St. Louis, MO, USA).

### 2.2. Collection of Saliva

The participants for supplying saliva were 28 years old with good oral hygiene and good general health. The subjects were asked to not eat or drink two hours before sampling to avoid any other factors influencing the component of saliva. A total of 0.5 mL of saliva was taken and then placed in a 1.5 mL EP tube. After centrifuging (8000 rpm, 4 °C, 10 min), the supernatant was placed in another EP tube for RNA digestion.

### 2.3. Degradation of Yeast RNA by Saliva

Firstly, 2.0 μL of pH 2.2 (or other pH) Na_2_HPO_4_ (0.73 mM)/citric acid (9.6 mM) buffer, 2.0 μL of yeast RNA (16 mg/mL), 5.0 μL centrifuged saliva, and 1.0 μL RNase inhibitor (40 U/μL) were added to 10 μL of pure water. The reaction (20 μL in total) was carried out at 37 °C for 1 h. For other pHs, the ratio between Na_2_HPO_4_ and citric acid was changed to adjust pH. Then, an equal volume of RNA extraction agent (pH ≤ 5.0) was added to the above reaction system (for removing proteins from the reaction system). After mixing well, the mixed liquid was centrifuged (12,000 rpm, 4 °C, 10 min), and the upper aqueous solution was transferred to another 200 μL EP tube. Finally, an equal volume of chloroform/isoamyl alcohol (24:1) was added, and the centrifugation and separation were repeated.

### 2.4. Degradation of Nucleic Acids by DNase II

For a typical reaction, 2.0 μL of pH 2.2 (or other pH) Na_2_HPO_4_/citric acid buffer, 2.0 μL yeast RNA (16 mg/mL), 2.0 μL DNase II (5 U/μL), and 1.0 μL RNase inhibitor (40 U) were added to 13.0 μL pure water. The reaction (20 μL in total) was carried out at 37 °C for 0.5 h and treated as above to remove proteins. The digestion of salmon sperm DNA was carried out similarly to yeast RNA, except that 1.0 μL RNase inhibitor (40 U/μL) was replaced with pure water. In some cases, 4 μL of copper sulfate (0.025 M/0.05 M/0.25 M/0.5 M) was added to the above reaction (20 μL in total), and other conditions were the same as above (for proving whether copper ions can inhibit DNase II from degrading yeast RNA or salmon sperm DNA).

The digestion of transcribed RNA (see Supplemental Table S1 for the sequences) under weak conditions was carried out as follows. A total of 2.0 μL of pH 6.0 Na_2_HPO_4_/citric acid buffer, 2.0 μL R-60 (20 μM, transcribed by two purchased DNA sequences), 2.0 μL DNase II (0.2 U/μL), and 1.0 μL RNase inhibitor (40 U/μL) were added to 13.0 μL pure water. The reaction (20 μL in total) was carried out at 37 °C for various time intervals and treated as above to remove proteins. Another nucleic acid degradation reaction was similarly carried out. For nucleic acids with a loop or a gap structure, before digestion by DNase II, the samples were heated to 60 °C for 10 min, followed by cooling gradually (0.1 °C/s) to 25 °C and kept for 10 min (annealing for hybridization).

R-60 was transcribed as follows: Firstly, the DNA template for transcription was prepared. A total of 1 μL of the template strand M-46 (10 μM), 1 μL coding strand B-51 (10 μM), and 1 μL 10× Klenow Buffer were added to 4.6 μL pure water. After being heated to 60 °C for 10 min, the solution was cooled gradually (0.1 °C/s) to 20 °C and kept for 10 min. Then, 2 μL dNTP (1 mM) and 0.4 μL Klenow DNA polymerase (2.5 U/μL) were added to the above reaction system. The reaction (10 μL in total) was carried out at 25 °C for 15 min. The Klenow was deactivated by incubation at 75 °C for 20 min. Transcription: 1 μL of the above double-stranded DNA template (0.5 μM), 1 μL each NTP (40 mM), 1 μL RNase inhibitor (40 U/μL), 10 μL GMP (40 mM), 2 μL 10× T7 RNAP Buffer, and 0.8 μL T7 RNAP (50 U/μL) were added to 1.2 μL pure water. The reaction (20 μL in total) was carried out at 37 °C for 40 min. The T7 RNAP was deactivated by incubation at 70 °C for 10 min.

### 2.5. Exonuclease Digestion of Products after RNA Digestion by DNase II

Digestion by exonuclease XRN-1: 3.0 µL of the above reaction system (R-60 digested by DNase II), 1.0 µL 10× XRN-1 buffer (pH 7.9 at 25 °C, 1.0 M NaCl, 0.5 M Tris-HCl, 0.1 M MgCl_2_, 10 mM DTT), 0.5 µL RNase inhibitor (40 U), and 2 µL XRN-1 (1 U/µL) were added to 3.5 µL pure water. The reaction (10 μL in total) was carried out at 37 °C for 5 h. XRN-1 was deactivated by incubation at 70 °C for 10 min.

Digestion by exonuclease EXO T: 3.0 µL of the above reaction system, 1.0 µL 10× EXO T reaction buffer (pH 7.9 at 25 °C, 0.5 M Potassium Acetate, 20 mM Tris-acetate, 100 mM Magnesium Acetate, 10 mM DTT), 0.5 µL RNase inhibitor (40 U/µL), and 2 µL EXO T (5 U/µL) were added to 3.5 µL pure water. The reaction (10 μL in total) was carried out at 25 °C for 5 h. EXO T was deactivated by incubation at 65 °C for 20 min.

### 2.6. Dephosphorylation of RNA Products Digested by DNase II

A total of 2 µL of 10× Fast AP reaction buffer (pH 8.0 at 37 °C, 100 mM Tris-HCl, 50 mM MgCl_2_, 1 M KCl, 0.2% Triton X-100, and 1 mg/mL BSA), Fast AP (1 U/μL), and 1 µL RNase inhibitor (40 U/μL) were added to 15 µL RNA digested products. The reaction (20 μL in total) was carried out at 37 °C for 2 h. Fast AP was deactivated by incubation at 75 °C for 5 min.

### 2.7. Phosphorylation of Purchased DNA

A total of 2 µL 10× T4 PNK reaction buffer (pH 7.0 at 25 °C, 700 mM Tris-HCl, 100 mM MgCl_2_, 50 mM DTT), 8 µL ssDNA (100 μM), 0.5 µL ATP (10 mM), and 1 µL T4 PNK (10 U/μL) were added to 8.5 µL pure water. The reaction (20 μL in total) was carried out at 37 °C for 8 h. T4 PNK was deactivated by incubation at 65 °C for 20 min.

### 2.8. Ligation of Products Digested by DNase II

For a typical reaction, 10.0 µL digested products (after dephosphorylation), 2 µL 10× T4 Dnl buffer, 2 µLTem54 (20 µM), and 0.5 µL RNase inhibitor (40 U/μL) were added to 1.5 µL pure water. After being heated to 85 °C for 15 s, the solution was cooled gradually (0.1 °C/s) to 65 °C and kept for 10 min, and then the solution was cooled gradually (0.1 °C/s) to 37 °C and kept for 10 min. Finally, 1.0 μL of Dna-41 (40 µM, phosphorylated) and RNase inhibitor (40 U/μL) and 2.0 μL T4 Dnl (5 U/μL) were added. The ligation reaction (20 μL in total) was carried out at 37 °C for 5 h. T4 Dnl was deactivated by incubation at 65 °C for 10 min. The ligation of other phosphorylated ssDNAs was carried out similarly as above.

## 3. Results

### 3.1. Efficient Degradation of RNA by DNase II under Acidic Conditions

**Figure 1:**
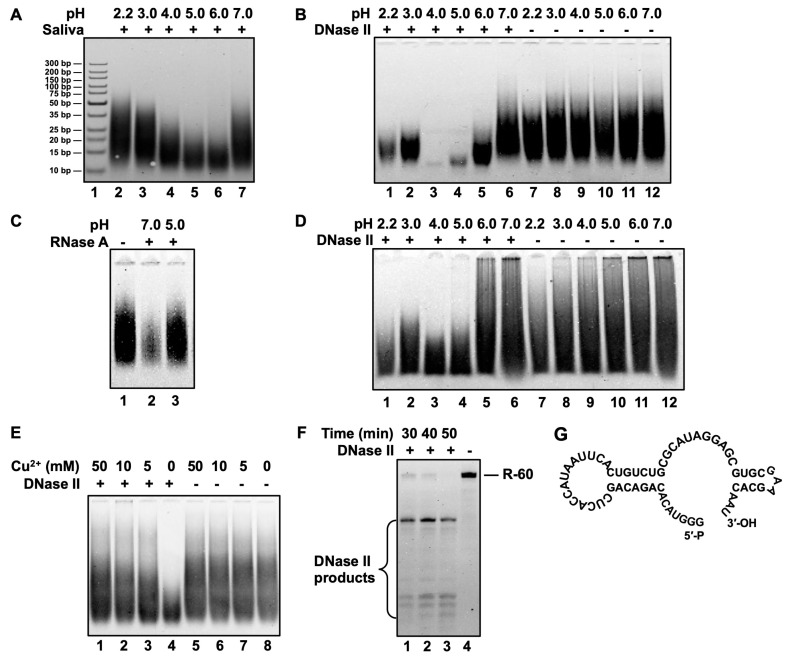
Digestion of RNA (or dsDNA) by saliva or DNase II under various pHs. (**A**) Digestion of rRNA by human saliva (mixture of 15 µL saliva and 5 µL Na_2_HPO_4_/citric acid buffer) at pH 3.0 or pH 4.0 for 4 h. (**B**) Digestion of rRNA by 0.5 U/µL DNase II at various pHs (pH 2.2, 3.0, 4.0, 5.0, 7.0) for 1.0 h. Other conditions for the following (**A**,**B**): 1.6 g/L yeast RNA, 2 U/µL RNase inhibitor, 37 °C. (**C**) Digestion of rRNA by RNase A. Conditions: 1.6 g/L RNA, 0.1 µg/mL RNase A, pH 8.0 (Lane 2) or pH 5.0 (Lane 5.0), 37 °C, 15 min. (**D**) Digestion of salmon sperm dsDNA by DNase II at various pHs. Conditions: 1.6 g/L DNA, 0.5 U/µL DNase II, 37 °C, 3 h. (**E**) Digestion of RNA in presence of copper ions. Conditions: 0.5 U/µL DNase II, pH 4.0, 5, 10, 50, or 100 mM of CuSO_4_, 37 °C, 0.5 h. (**F**) Digestion of R-60 (transcribed RNA) by DNase II. Conditions: 2 µM R-60, 0.02 U/µL DNase II, 2 U/µL RNase inhibitor, pH 6.0, 37 °C, 5, 15, or 30 min. For (**A**–**E**), 12% APGE; for (**F**), 6% dPAGE was used. For all experiments, Na_2_HPO_4_/citric acid buffer was used. (**G**) Secondary structure of R-60.

Considering DNase II shows (DNA digestion) activity only under weak acidic conditions (pH 3–6), and a substantial amount of DNase II was secreted in saliva as well as in the stomach [6,9,34], we hypothesize that DNase II secreted in saliva should digest the nucleic acid of food in the stomach. In food or living cells, RNA (about 85% is ribosome RNA, i.e., rRNA) is usually much more abundant than DNA (by about 10-fold). In addition, it is found that the digestion of DNA by DNase II was inhibited in the presence of RNA [18,19]. Accordingly, we suppose that DNase II may digest RNA. To confirm this, we first checked whether saliva could digest rRNA under acidic conditions. As shown in Figure 1A, in the presence of RNase inhibitor (for RNase A), rRNA becomes much shorter under pH 4–6 (Lanes 4–6), as compared with other pHs, indicating that saliva can digest rRNA under weak acidic conditions. Similar phenomena can also be observed for saliva from other volunteers, although the optimal pH varies to some extent (Supplemental Figure S1). This result prompted us to use DNase II to check whether it has RNA digestion activity. 

As shown in Figure 1B, rRNA is greatly digested by DNase II, especially at pHs 4.0 and 5.0 (Lanes 3 and 4). The digestion is not as efficient at pHs lower than 4.0 (see Lanes 6–8). It is noteworthy that only <20 mM Na_2_HPO_4_ is present in the buffer. Considering that saliva may contain DNase I and RNase A [6,9,34], and RNase A contamination may occur during RNA digestion by DNase II, RNase A inhibitor is added in the above experiments. It is well known that RNase A is active at pH 7.0–8.0 [35]. Figure 1C also shows that RNase A can only digest RNA under neutral pH (comparing Lane 2 with Lane 3) in the absence of an inhibitor. These results confirm that the RNA digestion shown in Figure 1A,B is not caused by RNase A. Under the same conditions, the digestion of salmon DNA (10–1000 bp dsDNA) by DNase II shows similar pH dependence (Figure 1D). At pH 2.2 (Lane 1), the bands become weaker to some extent, probably because slight depurination occurs [36]. Interestingly, DNase II digests RNA much more efficiently as compared with the digestion of DNA (comparing Figure 1B with Figure 1D). Accordingly, it can be concluded that DNase II indeed digests RNA under acidic conditions, i.e., DNase II in the saliva is secreted in the mouth, and it digests RNA in the stomach.

The RNA digestion activity of DNase II is further confirmed by using divalent copper ions (Figure 1E), based on the fact that its DNA digestion activity is specifically inhibited (but not RNase A) by copper ions [2,13,17]. As expected, copper ions as low as 5 mM greatly inhibit RNA digestion by DNase II (Lane 3, Figure 1E), further eliminating the possibility that RNA digestion is caused by RNase A contamination.

It has been reported that DNase II has some sequence specificity under weak conditions, especially for G- and A-rich sequences (e.g., AGA↓GGA). Accordingly, we designed an RNA sequence (R-60) as shown in Figure 1F to check whether RNA digestion by DNase II has similar sequence specificity. R-60 with two stable hairpin structures and an ssDNA part containing an AUAGGAG sequence was transcribed by T7 RNA polymerase using the dsDNA as the template attaching the T7 promoter. Under a very low concentration of DNase II (0.02 U/µL, only 1/25 of DNase II is used for experiments in Figure 1B) and at pH 6.0, a distinct product band was observed after 2.0 μM of R-60 was cleaved for 30 min (Lane 1, Figure 1F). As proven later, R-60 was cleaved at AUAGGAG. The left loop structure with a sequence of CACUUAAUACCACUC was cleaved with a lower activity. The dsRNA parts (two stems in hairpins) should be cleaved more difficultly probably because DNase II digests dsDNA much more difficultly than it digests ssDNA. When a higher concentration (0.04 U/µL) of DNase II is used, R-60 can be completely digested (Figure S2A). Again, shortening the cleavage time can also provide a clearer band due to specific cleavage (Figure S2B). These results show that DNase II can cleave RNA with similar patterns for cleaving DNA.

### 3.2. RNA Is Digested by DNase II Similarly to ssDNA

**Figure 2:**
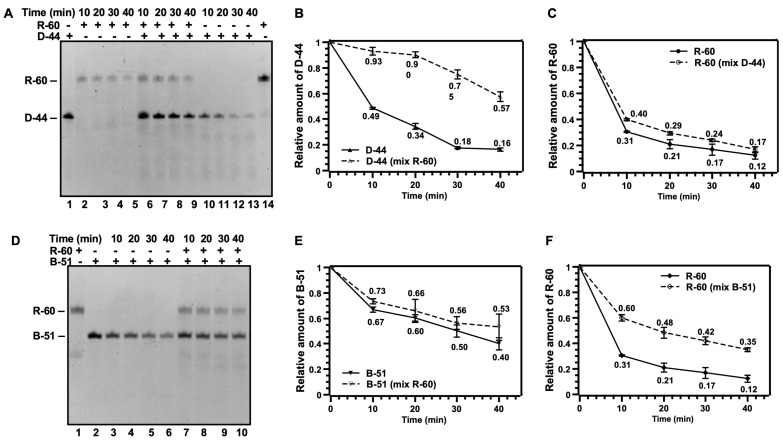
Digestion of RNA by DNase II in presence of ssDNA. Conditions: 2 µM ssDNA, 2 µM R-60, pH 6.0 (Na_2_HPO_4_-citric acid buffer), 2 U/µL RNase inhibitor, 0.02 U/µL DNase II, 37 °C for 10, 20, 30, or 40 min, analyzed by 12% dPAGE (8 M urea). (**A**) Electrophoresis analysis for digestion of R-60 (Lane 2–5), D-44 (Lane 11–13), and mixture of D-44 and R-60 (6–10) for various time intervals. Lane 1: D-44, Lane 14: R-60. (**B**) Quantitative analysis for digestion of D-44 in absence and presence of R-60. (**C**) Quantitative analysis for digestion of R-60 in absence and presence of D-44. Relative brightness of substrate bands in (**A**) is used. (**D**) Electrophoresis analysis for digestion of another ssDNA of B-51 (Lane 3–6) and mixture of B-51 and R-60 (7–10) for various time intervals. Lane 1: R-60, Lane 2: B-51. (**E**) Quantitative analysis for digestion of B-51 in absence and presence of R-60. (**F**) Quantitative analysis for digestion of R-60 in absence and presence of B-51. Relative brightness of substrate bands in (**A**,**D**) is used. D-44 has same sequence as 5′-part of R-60. B-51 contains T7 promoter sequence (19 nt) and 32 nt long sequence same as 5′-part of R-60.

For comparing the digestion activity of DNase II between RNA and DNA, as well as confirming the inhibition effect of DNA digestion in the presence of RNA, a DNA and RNA mixture was used as the substrate for DNase II’s digestion. First, 44 nt DNA (D-44) was designed to have the same sequence as the 44 nt long 5′-part of R-60. The shorter DNA was used in order to distinguish it from R-60 and their digestion products. After digestion by 0.02 U/µL DNase II (a weak condition) for 40 min, only a small amount of D-44 was left (Lane 13 in Figure 2A). Figure 2B shows the quantitative analysis of the data in Figure 2A, clearly showing that D-44 was gradually digested by DNase II. Interestingly, for the mixture of D-44 and R-60, only 43% of D-44 (57% left) was digested under the same conditions (Lane 9 in Figure 2A,B), indicating that the presence of R-60 inhibited the digestion of D-44. Meanwhile, about 83% of R-60 (17% left) was digested even in the presence of D-44 with a small inhibition effect (Lanes 6–9, Figure 2A,C).

Additionally, 51 nt ssDNA (B-51) containing a T7 promoter sequence (19 nt) and 32 nt long sequence the same as the 5′-part of R-60 was also used. After the digestion of the mixture of B-51 and R-60 for 40 min under the same conditions, 53% of B-51 was left (Lane 10 in Figure 2D,E), indicating that R-60 also inhibited the digestion of B-51 to a small extent (comparing Lanes 3–6 with Lanes 7–10). On the other hand, 35% of R-60 was left in the presence of B-51 (Lane 10, Figure 2D,F), which is quite more than that in the absence of B-51 (Figure 2F). This shows that digestion of R-60 was obviously inhibited by B-51. Interestingly, comparing Figure 2B,C with Figure 2E,F, the presence of R-60 decreased the digestion rate of D-44 more than that of B-51, and the presence of B-51 decreased the digestion rate of R-60 more than that of D-44. The lower digestion of B51 may be due to its lack of the ATAGGAG sequence, and its longer ssDNA 5′-part (26 nt) may bind DNase II more strongly than secondary structures in D-44 and R-60. These results demonstrate again that DNase II can bind and digest both DNA and RNA, although the digestion has sequence specificity to some extent under weak conditions. Obviously, RNA digestion by DNase II is faster than DNA digestion (comparing Figure 2B with Figure 2C).

### 3.3. Determination of Terminal Structure of RNA Cleavage Products

**Figure 3:**
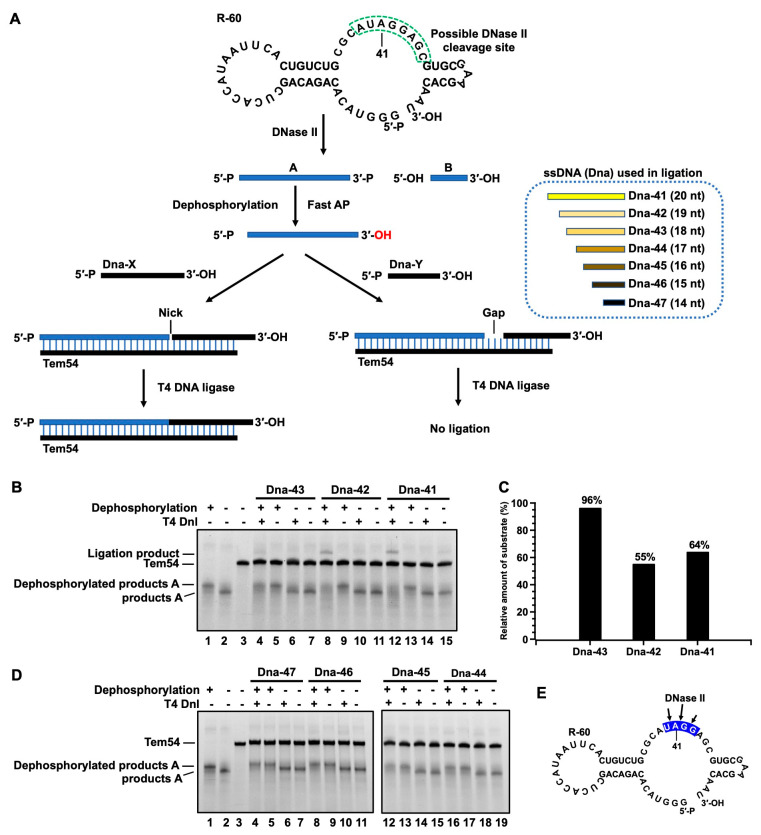
Analysis of cleavage sites of R-60 by DNase II and terminal structure of cleavage products. (**A**) Strategy diagram for analyzing by ligation of product A with ssDNA. If product A has 3′-PO_4_, ligation occurs only after dephosphorylation; cleaving sites can be determined by checking which ssDNA is ligated. (**B**,**D**) PAGE analysis of ligation results for various ssDNAs. Lane 1: product A after dephosphorylation, Lane 2: product A before dephosphorylation, Lane 3: Tem54. In (**B**), Lanes 4−7: Dna-43, Lanes 8−11, Dna-42, Lanes 12−15: Dna-41, Lanes 16−19: Dna-44. In (**D**), Lanes 4−7: Dna-47, Lanes 8−11, Dna-46, Lanes 12−15: Dna-45, Lanes 16−19: Dna-44. Conditions: dephosphorylated/non-dephosphorylated RNA cleavage product A, 2 µM Tem54, 2 µM ssDNA, 2 U/µL RNase inhibitor, 1.5 U/µL T4 Dnl, 37 °C for 4 h; 12% dPAGE (25% formamide and 7 M urea). (**C**) Quantitative analysis of cleavage product A based on (**A**). (**E**) Schematic illustration of RNA cleavage sites, using arrows to show cleavage sites and length of arrows to show strength.

To determine whether the RNA digestion products have 3′-PO_4_ and 5′-OH, which are the same as those in the DNA digestion by DNase II, the following experiments were designed to assign the cleaved products of R-60 (Figure 3A). Considering that DNase II has a preference to cut at the A- and G-rich sites [16], we hypothesized that DNase II cleaves at the ssDNA part (green dashed box in Figure 3A) containing the AUAGGAG sequence of R-60. To clarify the exact cleavage site, seven ssDNA oligos (Dna-41–Dna-47) were designed for ligation. Six nt adenines (A_6_) were attached to Dna-41–Dna-47 to avoid nonspecific ligation. Tem54 was used as the template to ligate cleaved RNA products and these ssDNA oligos (Dna-41–Dna-47). From the position of the bands of cleavage products in the electrophoresis gel (Figure S2), the main cleavage product A can be assigned about 40 nt in length and product B about 20 nt in length. After ligation, the ligated products should be 60 nt long for all the ssDNAs. T4 DNA ligase was used because it can ligate a DNA or RNA with a 5′-PO_4_ end to an RNA with 3′-OH by forming a nick [16]. Certainly, T4 DNA ligase cannot ligate a substrate with a 3′-PO_4_ end at the nick. If RNA products attach 5′-PO_4_, they can be ligated directly to a DNA with 3′-OH. If they have 3′-PO_4_, they can only be ligated after dephosphorylation, whereas non-dephosphorylated cleavage products cannot. 

The ligation experiment results are shown in Figure 3B,D. After dephosphorylation by Fast AP (an enzyme), a band with lower mobility appears (comparing Lane 1 with Lane 2 in Figure 3B), indicating that the dephosphorylation is successful. For Dna-42, as an example for ligation experiments (Lane 8−11, Figure 3B), a new band appears only after dephosphorylation and ligation (compare Lane 8 with Lane 10), and the band for product A (about 40 nt long) becomes weaker. Similar results are obtained for Dna-41 and Dna-43, although the ligation product for Dna-43 (Lane 4) is much weaker than that for Dna-41 (Lane 12). No ligation products can be observed for Dna-44, 45, 46, and 47 (Figure 3D and Figure S4). These results demonstrate that the cleavage products by DNase II have a 3′-PO_4_ (but not 3′-OH), and the cleavage sites are AU↓AGGAG, AUA↓GGAG, and AUAG↓GAG, respectively. The relative quantitative results for the ligation of product A are represented in Figure 3C. For Dna-42, 45% of product A is ligated and 36% for Dna-41. Accordingly, the cleavage efficiency is in the following order: AUA↓GGAG ≈ AU↓AGGAG > AUAG↓GAG (also shown in Figure 3E using arrows). It also shows that DNase II has a preference for cleaving the phosphodiester bond between A and G. 

This conclusion (product A has 3′-PO_4_, and product B has 5′-OH) is further confirmed by digestion using exonucleases XRN-1 and EXO T (Figure S3). XRN-1 is known to digest DNA or RNA from the 5′-PO_4_ end to the 3′-end [37], while EXO T digests from the 3′-OH single-stranded overhang to the 5′-end [38]. The digestion results show that XRN-1 can digest R-60 and product A, demonstrating that product A has a 5′-PO_4_ end (introduced during transcription by using GMP), and Product B has 5′-OH. EXO T could digest R-60 but not Product A, showing again that Product A has 3′-PO_4_.

### 3.4. DNase II Only Has Weak Sequence Specificity for Cleaving RNA and Can Cleave Most RNA Sequences Efficiently under Relatively Strong Conditions

**Figure 4:**
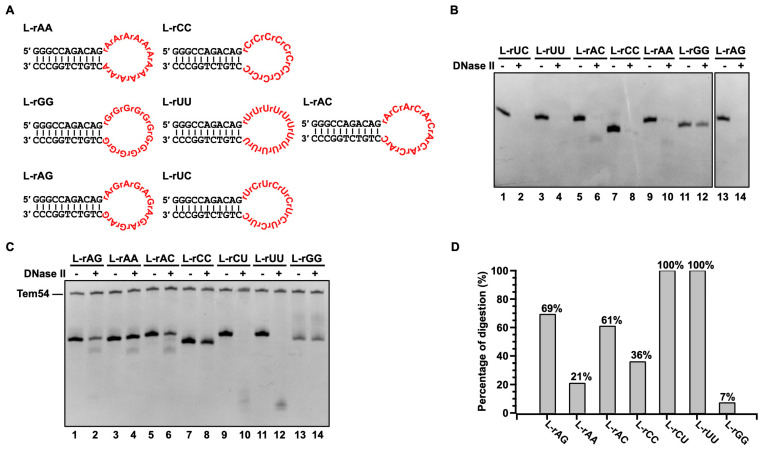
Sequence preferences for RNA digestion by DNase II. (**A**) Structure and sequences. (**B**) and (**C**) PAGE analysis of digestion results. For (**B**), stronger conditions of 0.04 U/µL DNase II, 40 min is used. For (**C**), weaker conditions of 0.02 U/µL DNase II, 10 min is used. Other conditions: 2 µM substrate, pH 6.0, 2 U/µL RNase inhibitor, 37 °C; 12% dPAGE (25% formamide and 7 M urea). For decreasing error caused by loading, ssDNA (Tem54) is mixed in loading buffer as inner standard. (**D**) Quantitative analysis of results in (**C**).

Previous studies have shown that DNase II is sensitive to the geometry of the DNA backbone (duplex or single-stranded) rather than the base sequence itself [39,40]. Here, we show that DNase II cleaves RNA with a similar pattern (Figure 3E and Figure S3). To further check the sequence specificity, substrates with a hairpin structure containing a loop were designed (Figure 4A). The loop has various RNA sequences composed of poly-pyrimidine, poly-purine, and alternating pyrimidine–purine bases. To observe the differences in digestion, relative weak conditions of 0.02 U/µL DNase II and 10 min are used (Figure 4C). As shown in Figure 4D, 100% of L-rUU and L-rCU, 69% of L-rAG, and 60% of L-rAC are consumed. For L-rCC, L-rAA, and L-rGG, the cleavage efficiency is relatively low. In summary, the cleavage efficiency is in the following order: L-rUU ≈ L-rUC > L-rAG > L-rAC > L-rCC > L-rAA > L-rGG (Figure 4D). For DNA with similar sequences, a similar order is obtained: L-AA ≈ L-TT ≈ L-CT > L-AG ≈ L-AC > L-CC > L-GG (Figure S5). The big difference between RNA and DNA is that poly-rA is digested difficultly, but poly-dA can be digested efficiently. These results prove again that RNA can be digested by DNase II using the same active center. All these sequences can be digested efficiently except for polyG by DNase II under relatively strong conditions of 0.04 U/µL DNase II and 40 min (Figure 4B).

Another interesting question is whether the DNA/RNA hybrid can be digested by DNase II. To check this, we used D-44a (an ssDNA complementary partly to R-60) which forms a hybrid with R-60 consisting of a 44 nt long DNA/RNA duplex part and a 16 nt long ssRNA overhang (Supplementary Figure S6). Interestingly, the digestion of this DNA/RNA hybrid was observed if the reaction time is long enough. When 0.2 U/µL DNase II was used, about 10% of D-44a was left after 60 min (Supplementary Figure S6A). When 0.02 U/µL DNase II was used, almost all D-44a and R-60 was cleaved after 6 h (Supplementary Figure S6B). The bands of D-44a and R-60 became weaker simultaneously, indicating that they were digested equally. Accordingly, it can be concluded that the DNA/RNA hybrid part is digested at least not less efficiently than the 16 nt long RNA overhang.

## 4. Discussion

The functions of DNase II have puzzled researchers for more than 50 years, and they are becoming increasingly interested in studying DNase II because it has great biological significance. It seems that we must study it from another perspective. Considering that nucleic acids have to be recycled in an organism as well as in the environment, we investigate DNase II from the perspective of nutrition, i.e., digesting nucleic acid for reuse. As shown in Figure 1, it is clearly shown that DNase II can digest both RNA and DNA under weak acid conditions. Obviously, for functions related to replication, transcription, and translation, nucleases with activity only for DNA digestion or RNA digestion are usually required. For the catabolism of nucleic acids as nutrients, activity to digest both DNA and RNA is reasonable and advantageous. Accordingly, we propose that DNase II is a widely existing nuclease with a basic function for digesting both DNA and RNA, either within a cell or in ingested food. This function is also reflected by the suggestion that DNase II is secreted in the mouth and functions in the stomach. 

It is well established that nucleases in saliva predominantly include hRNase-1 (a direct homolog of bovine pancreatic RNase A) [41], DNase I, and DNase II [6,9,34]. It is reasonable that all DNA and RNA can be elementarily digested in the mouth under neutral conditions (about pH 7) by DNase I and hRNase-1, respectively. The leftover DNA and RNA are further digested in the stomach by DNase II. It is noteworthy that the time for food kept in the stomach (several hours) is much longer than in the mouth (minute level), so the digestion of nucleic acid occurs mainly in the stomach or intestine. Consistent with this proposal, our research shows that saliva can digest RNA only under acidic conditions in the presence of the RNase inhibitor which blocks RNA digestion by hRNase-1 (Figure 1). Obviously, the only possibility left is that RNA degradation in saliva under acidic conditions is caused by DNase II. This is further proven by the fact that the addition of an RNase inhibitor does not block RNA digestion, showing that it is not caused by the contamination of RNase A (or hRNase-1) [42]. Additionally, the inhibition of RNA degradation by copper ions further proves that DNase II indeed digests RNA efficiently (Figure 1E).

The digestion results of the mixture of DNA and RNA clearly show that DNase II digests both RNA and DNA (Figure 2). RNA digestion is even more efficient than DNA digestion by DNase II. This result can explain why the addition of RNA can inhibit DNase II’s DNA digestion activity [18,19]. When RNA and DNA coexist, DNase II preferentially digests RNA (or the sequence to be more easily digested), thereby reducing the efficiency of DNA degradation. Copper ions can also inhibit RNA digestion by DNase II (Figure 1E), and the digestion of DNA and RNA has similar sequence preference (Figure 3 and Figure 4), indicating that they are digested at the same catalytic activity center. Thus, the mechanisms by which DNase II catalyzes the degradation of RNA and DNA may be identical, and the end structures of both enzymatic cleavage products should be the same, i.e., 3′-PO_4_ (5′-OH), which is evidenced by this study (Figure 3).

The data also show that RNA digestion by DNase II is very efficient and has only weak sequence dependence under a relatively high concentration (0.04 U/µL). Studies showed that nucleases that do not require divalent cations for catalyzing substrate degradation are affected to some extent by the substrate structure, such as base stacking but not sequences [43,44,45,46]. This also corresponds to its function of digesting nucleic acids. In addition, the digestion of a DNA/RNA hybrid (Figure S6), dsDNA, and RNA with a secondary structure (e.g., R-60) can be carried out at a normal concentration level of DNase II (0.1−1.0 U/µL), demonstrating that it is a powerful nuclease. 

**Figure 5:**
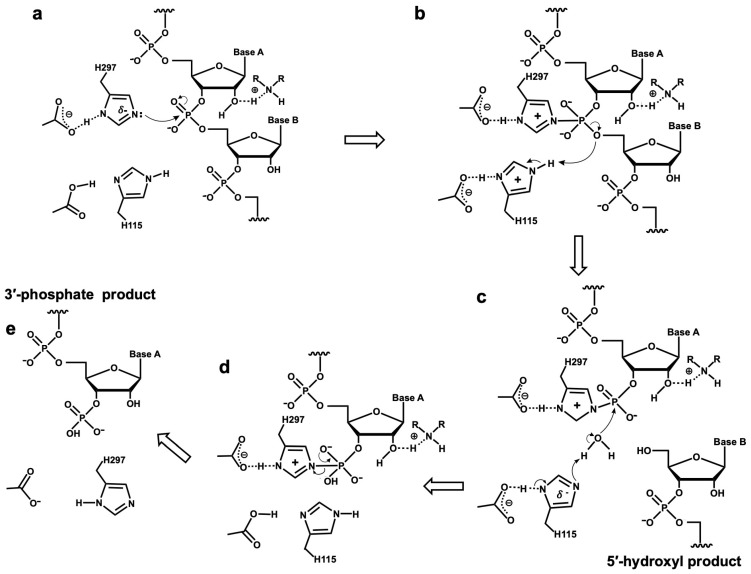
Mechanism for RNA cleavage catalyzed by DNase II. (**a**)→(**b**): His297 of DNase II is covalently bound to phosphodiester bond of RNA; (**b**)→(**c**): His115 of DNase II activates water molecule, which attacks phosphorus atom and releases RNA product attaching 5′-hydroxyl; (**c**)→(**d**): formation of intermediate; (**d**)→(**e**): His297 is dissociated from phosphate group, and RNA product attaching 5′-hydroxyl is released.

The mechanism for the DNase II catalysis of RNA digestion is proposed based on the known mechanism of DNase II’s DNA digestion. It is known that DNase II interacts with DNA through specific amino acid residues in the catalytic domain to achieve DNA cleavage [47]. Histidine residues are crucial for forming the phosphatase intermediate and subsequently activating water molecules to attack the covalently bonded phosphodiester bond [48,49,50]. The mechanism of porcine DNase II catalyzing DNA degradation is as follows: First, His297 acts as an electron donor to attack the phosphorus atom and form a covalent intermediate with the phosphate group, while His115 provides a proton for the cleavage of the P-O bond, releasing the cleaved DNA (3′ side of the substrate) with a 5′-hydroxyl end. Subsequently, His115 activates a water molecule, and the deprotonated water attacks the phosphorus atom, leading to a conformational change in the covalent intermediate, resulting in the cleavage of the N-P bond and the release of the cleaved DNA (5′ side of the substrate) with a 3′-phosphate end [51]. Considering that the structural difference between RNA and DNA lies in the 2′-OH on the pentose sugar, it is known that 2′-OH is catalyzed by the His12 of RNase A attacking the phosphorus atom of the phosphate group during RNA degradation by RNase A, forming a 2′,3′-cyclic phosphate, followed by the hydrolysis of the 2′,3′-cyclic phosphate intermediate by His12 and His119, generating 3′-phosphate and a small amount of 2′-phosphate products [52]. Therefore, if 2′-OH participates in the catalytic reaction of DNase II in RNA cleavage, it would hinder the binding of DNase II’s His297 to the phosphodiester bond of RNA to affect the release of 3′-phosphate products. Furthermore, since the lysine residues of DNase II are positioned near the catalytic active center [17,47], it is highly probable that the 2′-OH group of RNA binds to the amino group of lysine. This binding would prevent the 2′-OH group from attacking the phosphorus atom of the phosphate group and forming 2′,3′-cyclic phosphate. Based on the above analysis and previously reported results, the mechanism of the DNase II catalysis of RNA cleavage is proposed, as shown in Figure 5. Basically, it is similar to digesting DNA. The only difference is as follows: the 2′-OH of RNA binds to a lysine residue of DNase II, and thus, this 2′-OH is unable to attack phosphorus atoms on the phosphodiester bond of the substrate (Figure 5b,d). This may also be the reason that RNA is more easily digested by DNase II in some cases, i.e., DNase II binds to RNA more strongly than it binds to ssDNA. Future research should focus on clarifying the binding complex of DNase II and RNA and on understanding how RNA structure and base composition affect RNA digestion by DNase II to elucidate its mechanism.

**Figure 6:**
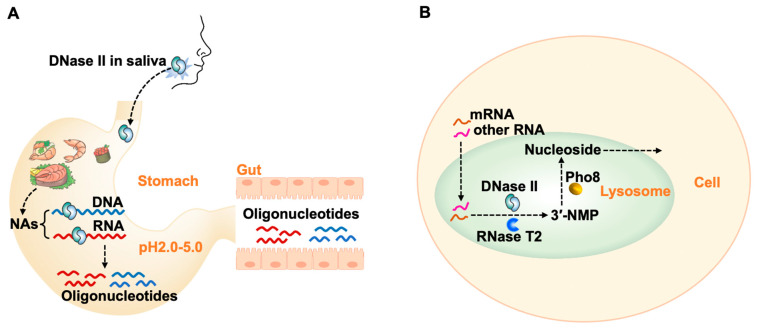
Proposed functions of DNase II. (**A**) Digestion of exogenous nucleic acids in digestive tract. DNase II (secreted in saliva and stomach) digests DNA and RNA in stomach into oligonucleotides. These oligonucleotides are further broken down or directly absorbed in small intestine. (**B**) Digestion of nucleic acids (mainly RNA) used in lysosomes to oligonucleotides. These oligonucleotides are further broken down into 3′-mononucleotides by RNase T2 and ultimately into nucleosides by Pho8. Nucleosides are then transported out of lysosome for cellular reuse [54,55].

According to the above results and discussion, the biological functions of DNase II can be summarized as digesting nucleic acids in food and transcribed RNA in cells (Figure 6). This may explain why DNase II is widely distributed in the lysosomes of various tissues and cells for recycling intracellular nucleic acids and why saliva secretes DNase II to utilize exogenous nucleic acids (DNA and RNA in the digestive tract) as nutrients. Previous research suggests that DNA oligonucleotides can be absorbed by intestinal epithelial cells via binding proteins [53] and can reach various organs and tissues through the bloodstream, including the hepatic portal circulation. mRNA, rRNA, and other cytoplasmic RNAs in the cytoplasm can be transported into lysosomes. Within lysosomes, DNase II, RNase T2, and Pho8 work together to digest these nucleic acids (mainly RNA) into nucleosides [54,55,56,57], which serve as substrates for the salvage synthesis of mononucleotides. The hydrolysis products by DNase II have a 3′-phosphate terminal structure, which cannot be used as substrates by enzymes such as ligases and polymerases and hence cannot participate directly in biological processes such as DNA replication. It is speculated that nucleic acid hydrolysis products with 3′-phosphate are ideal materials for forming a pool for supplying nucleotides. When nucleotides are deficient, short RNA or nucleotides with 3′-phosphate are transformed into nucleotides with 5′-phosphate.

## 5. Conclusions 

In conclusion, this study demonstrates that DNase II can digest RNA, expanding our understanding of DNase II’s activity. DNase II is a versatile nuclease capable of degrading both DNA and RNA, so we suggest that it should be redesignated as mammalian DNase II (RNase). Our results underscore the significant biological role of DNase II as a nuclease to digest nucleic acids as nutrients, either in the digestive tract or in the lysosome within a cell. These results also provide a theoretical foundation for the use of nucleic acids as universal nutrients by organisms.

## Data Availability

All datasets are presented in the main manuscript as well as in the supporting file, and there are no additional data to be deposited in any dataset.

## Supplementary Materials

The following supporting information can be downloaded at https://www.mdpi.com/article/10.3390/cells13181525/s1, Figure S1: Digestion of rRNA by other volunteers’ saliva (NO.1–NO.4) at various pHs (pH 2.2, 3.0, 4.0, 5.0, 7.0). Figure S2: Transcriptional ssRNA digested by DNase II under acidic conditions. Figure S3: Study on terminal structure of digested RNA products. Figure S4: Strategy for ligating product A with ssDNA to clarify end structure and cleavage sites. Figure S5: Digestion of DNA with various sequences in loop by DNase II. Figure S6: Digestion of DNA/RNA hybrid by DNase II. Table S1: Sequences used in this study. Table S2: Sequences used for ligation.
