# Supplementary material for: DNase II Can Efficiently Digest RNA and Needs to Be Redefined as a Nuclease
Source: Cells. 2024 Sep 11;13(18):1525. doi: 10.3390/cells13181525 (PMC11430429; doi:10.3390/cells13181525)
Supplement: Supplementary file 1 [file cells-13-01525-s001.zip › cells-3185830-Supplementary material.pdf]

Supplementary material to

DNase II Can Efficiently Digest RNA and Needs to Be Redefined as a Nuclease

Jingyun Zhuang [1], Xinmei Du [1], Kehan Liu [1], Jing Hao [1], Haoyu Wang [1], Ran An [1,2,*] and Xingguo Liang [1,2,*]

1 State Key Laboratory of Marine Food Processing & Safety Control, College of Food Science and Engineering, Ocean University of China, Qingdao 266404, China

2 Laboratory for Marine Drugs and Bioproducts, Qingdao Marine Science and Technology Center, Qingdao 266404, China

* Correspondence: ar@ouc.edu.cn (R.A.); liangxg@ouc.edu.cn (X.L.)

Table S1. Sequences used for digestion by DNase II.

Name	Sequence (5′→3′)	Length（nt）
B-51	GCTAATACGACTCACTATAGGGTACACAGACAGCTCACCA TAATTCACTGT	51
M-46	ATTGTGCTTCGCACGCTCCTATGCGCAGACAGTGAATTATG GTGAG	46
R-60	GGGUACACAGACAGCUCACCAUAAUUCACUGUCUGCGCA UAGGAGCGUGCGAAGCACAAU	60
D-44	GGGTACACAGACAGCTCACCATAATTCACTGTCTGTGCATA GGA	44
D-44a	TCCTATGCGCAGACAGTGAATTATGGTGAGCTGTCTGTGTA CCC	44
L-AA	GGGCCAGACAGAAAAAAAAAAAACTGTCTGGCCC	34
L-CT	GGGCCAGACAGCTCTCTCTCTCTGTCTGGCCC	34
L-CC	GGGCCAGACAGCCCCCCCCCCCCTGTCTGGCCC	34
L-GG	GGGCCAGACAGGGGGGGGGGGGCTGTCTGGCCC	34
L-TT	GGGCCAGACAGTTTTTTTTTTTCTGTCTGGCCC	34
L-AG	GGGCCAGACAGAGAGAGAGAGAGCTGTCTGGCCC	34
L-AC	GGGCCAGACAGACACACACACACTGTCTGGCCC	34
L-rAA	GGGCCAGACAGrArArArArArArArArArArArACTGTCTGGCCC	34
L-rCC	GGGCCAGACAGrCrCrCrCrCrCrCrCrCrCrCrCTGTCTGGCCC	34
L-rGG	GGGCCAGACAGrGrGrGrGrGrGrGrGrGrGrGCTGTCTGGCCC	34
L-rUU	GGGCCAGACAGrUrUrUrUrUrUrUrUrUrUrUCTGTCTGGCCC	34
L-rAG	GGGCCAGACAGrArGrArGrArGrArGrArGrArGCTGTCTGGCCC	34
L-rUC	GGGCCAGACAGrUrCrUrCrUrCrUrCrUrCrUrCCTGTCTGGCCC	34
L-rAC	GGGCCAGACAGrArCrArCrArCrArCrArCrArCCTGTCTGGCCC	34

Table S2. Sequences used for ligation.

Name	Sequence (5′→3′)	Length（nt）
Tem54	CAGTCAGTGCTCCTATGCGCAGACAGTGAATTATGGTG AGCTGTCTGTGTACCC	54
Dna-41	AGGAGCACTGACTGAAAAAA	20
Dna-42	GGAGCACTGACTGAAAAAA	19
Dna-43	GAGCACTGACTGAAAAAA	18
Dna-44	AGCACTGACTGAAAAAA	17
Dna-45	GCACTGACTGAAAAAA	16
Dna-46	CACTGACTGAAAAAA	15
Dna-47	ACTGACTGAAAAAA	14

Figure S1.

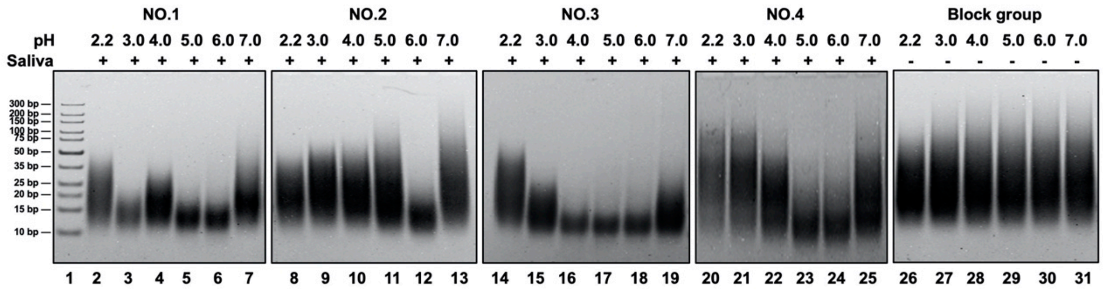

Figure S1. Digestion of rRNA by other volunteer's saliva (NO.1-NO.4) at various pHs (pH2.2, 3.0, 4.0, 5.0, 7.0). Conditions: 1.6 g/L yeast RNA, 2 U/μL RNase Inhibitor, 37°C for 2 h. For all experiments, Na_2HPO_4-citric acid and 10% of electrophoresis were used.

Figure S2.

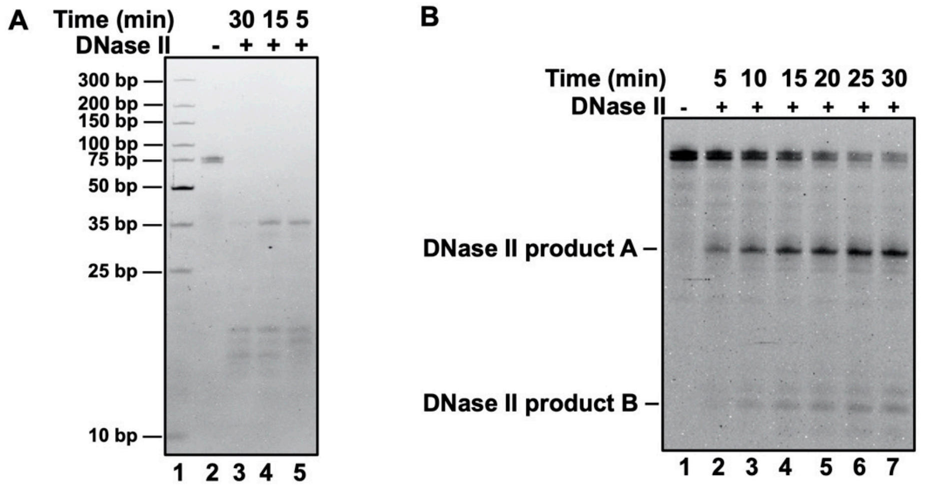

Figure S2. Digestion of transcriptional ssRNA (R-60) by DNase II under acidic conditions. (A) 1 μM R-60 was digested by 0.04 U/μL DNase II. Lane 1: ULR, Lane 2: R-60, Lane 3-5: R-60 digested for 30, 15, 5 min. (B) 2 μM R-60 was digested by 0.02 U/μL DNase II. Lane 1: R-60, Lanes 2-7: R-60 digested for 5, 10, 15, 20, 25, 30 min. Other conditions for (A) and (B): pH6.0 Na_2HPO_4-citrate buffer, 2.0 U/μL RNase Inhibitor, 37°C. For all experiments, 12% dPAGEs (8 M urea) were used.

Figure S3.

A

	DNase II products	Terminal structure		EXO T	XRN-1
Hypothesis 1	A	5′-PO_4	3′-PO_4	×	√
	B	5′-OH	3′-OH	√	×
Hypothesis 2	A	5′-PO_4	3′-OH	√	√
	B	5′-PO_4	3′-OH	√	√

- "√"show that DNase II products could be cleaved by EXO T or XRN-1;
- "×" show that DNase II products could [not be cleaved by EXO T or XRN-1
- The blue words represents the terminal structure formed at the cleavage sit after DNase II digestion.

B

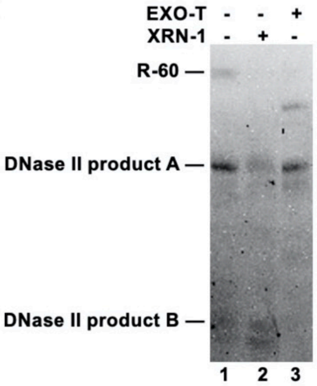

Figure S3. Digestion of cleaved product of R-60 by exonucleases to determine its terminal structure. (A) Explanation for digestion results by XRN-1 and EXO T; (B) PAGE analysis for RNA products cleaved by XRN-1 and EXO T. Lane 1: RNA products, Lane 2: XRN-1; Lane 3: EXO T. Conditions: 1 μM R-60 products, 0.2 U/μL XRN-1, 1× NEBuffer™ 3, 2 U/μL RNase Inhibitor, 37°C for 4 h, inactivate at 70°C for 10 min; Lane 3: 1 μM R-60 product, 1 U/μL EXO T, 1× NEBuffer™ 4, 2 U/μL RNase Inhibitor, 25°C for 4 h, inactivate at 65°C for 20 min. 12% of electrophoresis (8 M urea) were used.

Figure S4.

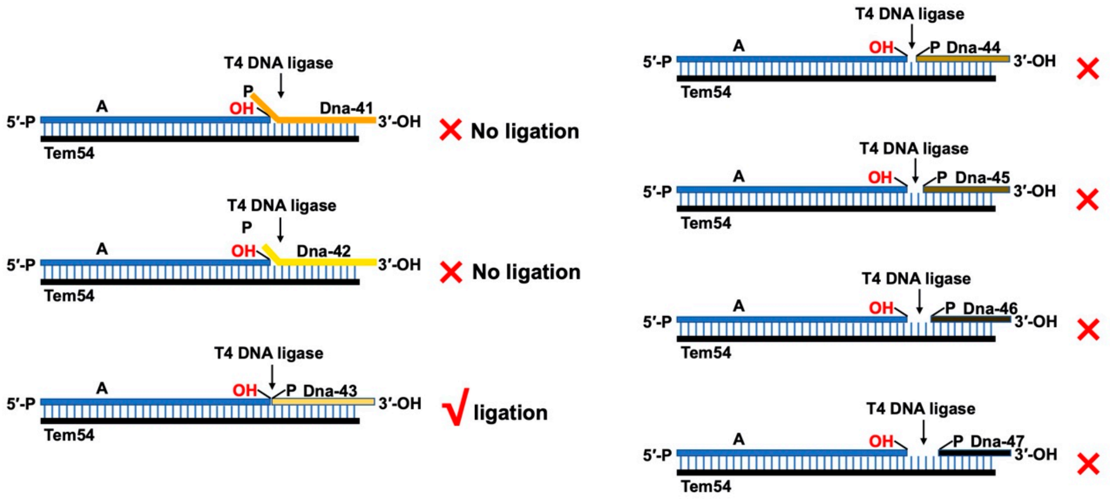

Figure S4. Supplementary strategy diagram for ligating DNase II product A with ssDNA. Product A: supposing the length of A is 40 nt. ssDNA: Dna-41, Dna-42, Dna-43, Dna-44, Dna-45, Dna-46 and Dna-47 (designed according to the possible DNase II cleavage sites of R-60).

Figure S5.

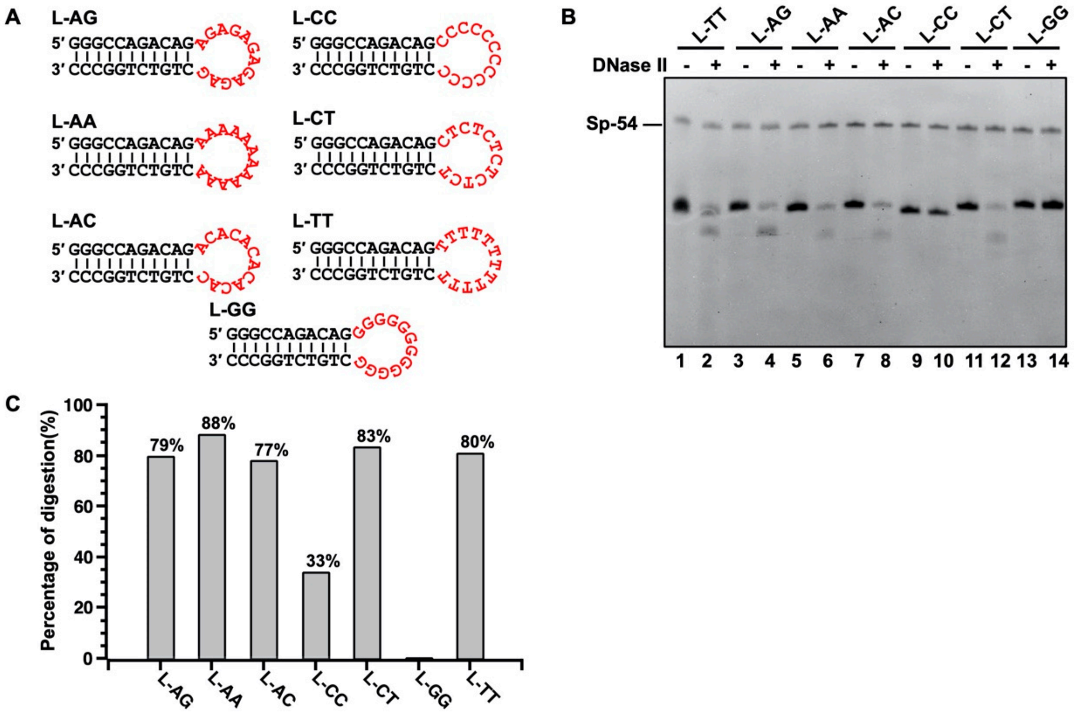

Figure S5. Digestion of DNA with various sequences at the loop by DNase II. (A) Secondary structure of DNA sequences. (B) PAGE analysis of digested products. Lanes 1, 2: L-AG; Lanes 3, 4: L-AA; Lanes 5, 6: L-AC; Lanes 7, 8: L-CT; Lanes 9, 10: L-CC; Lanes 11, 14: L-GT; Lanes 12, 13: L-TT. Conditions: 2 μM ssDNA, pH6.0 Na_2HPO_4-citrate buffer, 0.02 U/μL DNase II, 37°C for 20 min. Electrophoresis: 12% formamide. For decreasing the error caused by loading, a ssDNA (Tem54) is mixed in the loading buffer as an inner standard. (C) Quantitative analysis of data in (B). Image Lab software was used for relative quantification of substrate bands in (B).

Figure S6.

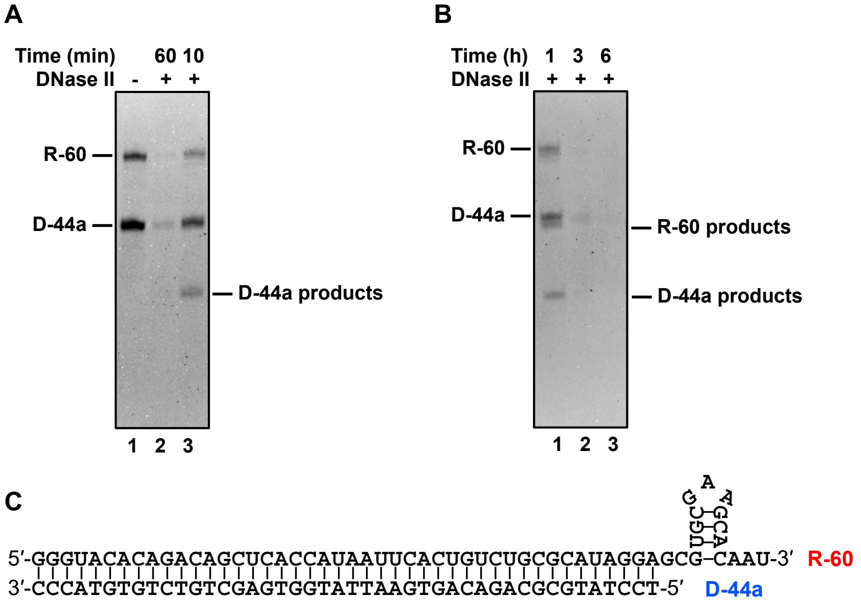

Figure S6. Digestion of DNA/RNA hybrid by DNase II**.** Electrophoresis analysis for digestion of DNA/RNA hybrid. Lanes 1: D-44a/R-60 hybrid. Condition: 2 μM D-44a, 2 μM R-60, pH6.0 (Na_2HPO_4-citric acid buffer), 2 U/μL RNase Inhibitor, 0.2 U/μL DNase II (A), 0.02 U/μL DNase II (B), 37°C, analyzed by 12% of dPAGE (25% formamide and 7 M urea). (C) Structure diagram of D-44a/R-60 hybrid. D-44a is 44-nt-long ssDNA. D-44a (an ssDNA complementary partly to R-60) which forms with R-60 a hybrid consisting of a 44-nt-long DNA/RNA duplex part and a 16-nt-long ssRNA overhang.
